# Comparison of Two Methods of RNA Extraction from Formalin-Fixed Paraffin-Embedded Tissue Specimens

**DOI:** 10.1155/2014/151724

**Published:** 2014-07-03

**Authors:** Gisele Rodrigues Gouveia, Suzete Cleusa Ferreira, Jerenice Esdras Ferreira, Sheila Aparecida Coelho Siqueira, Juliana Pereira

**Affiliations:** ^1^University of São Paulo's Medical School (FMUSP), 05403-000 São Paulo, SP, Brazil; ^2^Hospital das Clínicas da FMUSP, Laboratório de Imunopatologia, Avenida Doutor Enéas Carvalho de Aguiar, 155/1° Andar, Cerqueira César, 05403-000 São Paulo, SP, Brazil; ^3^FMUSP, São Paulo's Blood Center/Fundação Pró-Sangue's, Molecular Biology Department, 05403-000 São Paulo, SP, Brazil; ^4^FMUSP's Hospital das Clínicas (HC) Pathology Service, 05403-000 São Paulo, SP, Brazil; ^5^FMUSP, State of São Paulo Cancer Institute (ICESP), HC/FMUSP Central Institute (IC) Hematology Service, 05403-000 São Paulo, SP, Brazil

## Abstract

The present study aimed to compare two different methods of extracting RNA from formalin-fixed paraffin-embedded (FFPE) specimens of diffuse large B-cell lymphoma (DLBCL). We further aimed to identify possible influences of variables—such as tissue size, duration of paraffin block storage, fixative type, primers used for cDNA synthesis, and endogenous genes tested—on the success of amplification from the samples. Both tested protocols used the same commercial kit for RNA extraction (the RecoverAll Total Nucleic Acid Isolation Optimized for FFPE Samples from Ambion). However, the second protocol included an additional step of washing with saline buffer just after sample rehydration. Following each protocol, we compared the RNA amount and purity and the amplification success as evaluated by standard PCR and real-time PCR. The results revealed that the extra washing step added to the RNA extraction process resulted in significantly improved RNA quantity and quality and improved success of amplification from paraffin-embedded specimens.

## 1. Introduction

Assay of formalin-fixed paraffin-embedded (FFPE) tissue samples is a standard method for pathology examination as it is cost effective and ideal for preserving cell morphology [1]. However, molecular biology analyses are now frequently used to investigate many diseases, with the results translated to clinical practice, making it increasingly important to resolve several issues related to RNA extraction from FFPE samples [2, 3]. It is known that formalin may modify the structure and the chemical rearrangement of nucleic acids, particularly of RNA; therefore, RNA extracted from FFPE material may be of low quality and quantity [4, 5]. Since the first successful RNA extraction from FFPE materials, many protocols have been tested with the aim of reversing formalin-induced damage [2, 3, 5–8]. However, no investigation has yet identified the main step that interferes with the whole process. Furthermore, there is no consensus regarding the best protocol for RNA extraction from FFPE samples.

In attempts to increase the quality of RNA collected from paraffin-embedded materials, some researchers have tried replacing the formalin with other tissue fixatives—including Bouin's solution, Carnoy's fixative, acetone, alcohol, or the HEPES glutamic acid buffer-mediated organic solvent protection effect (HOPE) fixation—for pathology analyses to try to increase the quality of RNA collected from paraffin-embedded materials [5, 6]. However, these fixatives are related to tissue artifacts that may hinder both the histological tests and immunohistochemistry staining [5].

In the present study, we compare two different methods of extracting RNA from FFPE samples. We additionally investigate the main factors that may interfere with successful amplification, such as tissue size, paraffin block storage time, fixative type, and primers for complementary DNA synthesis, and the endogenous genes tested.

## 2. Material and Methods

Our study material included 83 samples from patients with diffuse large B-cell lymphoma from the archives of the Division of Pathology, Hospital das Clínicas, Faculty of Medicine, University of São Paulo. These samples were subjected to two protocols for RNA extraction, followed by cDNA synthesis, standard PCR, and real-time PCR, as described below. The summary of these results is shown in Table 1.

### 2.1. RNA Extraction (Protocol 1)

RNA extraction was performed as previously described [7], using the RecoverAll Total Nucleic Acid Isolation Optimized for FFPE Samples kit (Ambion Inc., Austin, Texas, USA). First, the samples were deparaffinized by addition of 1.0 mL xylene (Invitrogen, UK), followed by incubation for 5 minutes at 50°C, and centrifugation for 5 minutes at maximum speed. Next, the supernatant was discarded, and the pellet was washed twice with 1.0 mL absolute ethanol for rehydration. The proteins were degraded with 200 *μ*L digestion buffer and 5 *μ*L protease, followed by incubation for 15 minutes at 50°C and for 15 minutes at 80°C. Subsequently, RNA was isolated by adding 790 *μ*L of buffer containing absolute ethanol, along with passage through a purification column. The column was then washed twice with a buffer from the kit, and DNase treatment was performed, followed by two additional washings. Finally, RNA was eluted in 60 *μ*L of elution buffer from the kit at room temperature (RT) according to the manufacturer's instructions.

### 2.2. RNA Extraction Modified (Protocol 2)

The sections were rehydrated with absolute ethyl alcohol, followed by two additional washing steps using 1.0 mL of 10% phosphate-buffered saline (PBS; pH 7.2). Then the samples were centrifuged for 5 minutes at maximum speed to remove any remaining contaminants. Next, the sections were dried at room temperature (RT) prior to RNA extraction using the RecoverAll Total Nucleic Acid Isolation Optimized for FFPE Samples kit (Ambion Inc., Austin, Texas, USA) as described in protocol 1.

### 2.3. RNA Measurement

RNA concentration and purity were assessed using NanoDrop equipment (NanoDrop Technologies Inc., Wilmington, DE). Sample absorbance was measured at 260 nm and 280 nm, and the 260/280 ratio was used to assess RNA purity. RNA purity was considered adequate when the 260/280 ratio was ≥1.9, as a lower ratio could indicate the presence of proteins, phenol, or other contaminants that typically show strong absorbance at 280 nm [9].

### 2.4. cDNA Synthesis

For cDNA synthesis, 2 *μ*L of random primer or oligo dT was added to 10 *μ*L of extracted RNA. The samples were then heated at 70°C for 10 minutes and then cooled for 5 minutes at 45°C. Next, we added 8 *μ*L of the mix solution (4 *μ*L 5x buffer, 1 *μ*L DL-dithiothreitol (DTT), 1 *μ*L phosphate deoxyribonucleotides (DNTP) 10x, 0.5 *μ*L super script, 0.5 *μ*L RNAse inhibitor, and 1 *μ*L DNase/RNase-free water) to each sample and incubated them overnight at 45°C. Finally, the samples were homogenized, incubated at 70°C for 10 minutes, and stored at −20°C.

### 2.5. PCR

Each reaction mixture included 4.5 *μ*L 10x buffer, 1.0 *μ*L 10 mM DNTP, 3.0 *μ*L 50 mM MgCl_2_, 1 *μ*L 10 pmol/*μ*L forward primer, 1 *μ*L 10 pmol/*μ*L reverse primer, 5 *μ*L cDNA, and 34.2 *μ*L DNase/RNase-free water. We analyzed the endogenous genes* GAPDH* and *β*-actin, and DNase/RNase-free water was used as a negative control. Amplification was performed using the Mastercycler gradient thermalcycler (Eppendorf) programmed to heat to 94°C for 10 minutes, followed by 35 cycles of 94°C for 45 seconds, 55°C for 45 seconds, and 72°C for 2 minutes, and a final extension at 72°C for 15 minutes. The results were assessed in 1% agarose gel stained with ethidium bromide. The reaction products were applied to the agarose gel with the loading buffer and with a 100-bp ladder as a marker and were run at 100 V, 60 mA, and 40 W for 40 minutes. The results were analyzed using the image acquisition system, model Gel-Doc EZ (Bio-Rad Laboratories Inc.).

### 2.6. Real-Time PCR

Real-time PCR was performed using the TaqMan Universal PCR Master Mix (Applied Biosystems, USA). For each reaction, 12.5 *μ*L of the 2x Master Mix was mixed with 1.25 *μ*L of the 20x primer, 5 *μ*L of the cDNA, and 6.25 *μ*L of DNase/RNase-free water. The endogenous genes* GAPDH*,* PRKG1*, and* ABL1* were analyzed, and the FAM-TAMRA probe (Applied Biosystems, USA) was used as a marker, and DNAse/RNAse-free water was used as a negative control. Amplification was performed using iCycler equipment (Bio-Rad, USA) with initial 10 minutes at 95°C, followed by 45 cycles of 95°C for 15 seconds and 60°C for 1 minute. The reading was performed using the FAM-490 probe.

### 2.7. Statistical Analysis

Statistical analysis was performed using the chi-square and Mann-Whitney tests and Epi Info CDC software (v. 6.04, 2010), with 5% being chosen as the level of statistical significance.

## 3. Results

Using RNA extracted via protocol 1, 44.7% of samples showed negative results for PCR amplification of both endogenous genes *β*-actin and* GAPDH*. PCR was successful in only 25% of samples with RNA levels of 10–50 ng/*μ*L, 37.5% of samples with 50–100 ng/*μ*L, and 70.4% of samples with >100 ng/*μ*L of RNA (*P* = 0.00025). RNA purity analyses based on A260/280 ratio showed that among samples with a ratio of 1.7–1.9 (which is considered ideal by the manufacturers) only 16.7% showed positive amplification. However, a ratio of >1.9 allowed successful amplification in 62.5% of the samples (*P* = 0.0000001).

**Figure 1:**
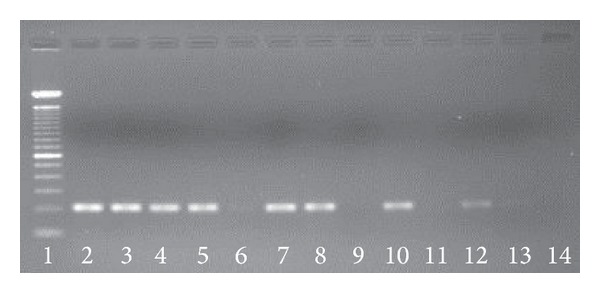
Standard PCR amplification of the *β*-actin gene. Amplification was detectable in the samples run in lanes 2–8, 10, and 12-13, with product corresponding to a 203-bp band. No detectable amplification occurred in the samples run in lanes 9 and 11. Lane 1 contains the 100-bp ladder, and lane 14 contains the negative control with DNase/RNase-free water rather than DNA.

**Figure 2:**
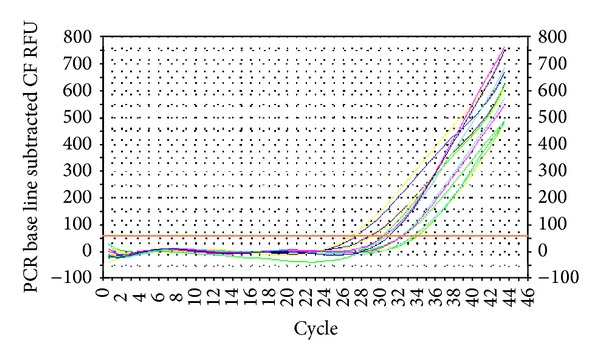
Curves showing positive real-time PCR amplification of the endogenous gene* PRKG1*. CF: curve fit; RFU: relative fluorescence units.

Amplification was observed in 16.7% of samples from tissue fragments of <1.0, in 71.4% from fragments of 1.0-2.0 cm, and in 75% of samples extracted from fragments larger than 2.0 cm (*P* = 0.034). Amplification levels were independent of tissue type, storage time, different primers used for cDNA synthesis (e.g., random primer or oligo dT), and the endogenous gene analyzed (i.e., *β*-actin,* GAPDH*,* ABL1*, and* PRKG1*). Amplification was successful in 87.5% of samples with formalin fixatives, compared to 17.6% for formaldehyde and 33.3% for Bouin's solution fixatives (*P* = 0.000018). The amplification success rate was 78.7% for standard PCR (Figure 1) and 95.7% among the samples for RT-PCR (Figure 2) (*P* = 0.045).

The statistical analysis demonstrated that the wash step with 10% PBS added after the sample rehydration stage—as performed in protocol 2—increased amplification success (*P* = 0.018). Protocol 2 resulted in the extraction of a higher quantity of RNA, despite using the same section quantities and thicknesses. No extraction via protocol 2 resulted in an RNA concentration of less than 50 ng/*μ*L. Among the samples with concentrations of 50–100 ng/*μ*L, amplification was successful from only 33.3%. On the other hand, amplification was successful from 89.3% of the samples with RNA concentrations of >100 ng/*μ*L. Among samples with an RNA purity of higher than a 1.9 ratio, amplification was successful from 83.9%. Among the samples fixed in formalin, 100% were amplified, compared to 80% of the samples fixed in Bouin's solution and 73.7% of the samples fixed in formaldehyde. Amplification was not influenced by tissue type or paraffin block age.

## 4. Discussion

Here we compared two protocols for RNA extraction from paraffin-embedded samples and evaluated different factors that might interfere with the amplification of genes from these samples. We introduced an additional step of washing with 10% PBS in protocol 2 because we hypothesized that the low amplification rate might be related to the presence of contaminants acting as PCR inhibitors. Our results demonstrated that the introduction of a washing step with PBS after sample rehydration significantly improved the quality of amplification of RNA extracted from paraffin-embedded material.

Formalin is the fixative most often used in pathological anatomy centers because it preserves cell morphology and allows immunohistochemical staining. In countries such as France and Canada, Bouin's solution fixative is also commonly used [10]. Both of these fixatives can interfere with molecular testing of paraffin-embedded materials [5]. As previously demonstrated, formalin can be metabolized into formic acid in the tissues, which in turn could hydrolyze the nucleic acids. However, the fixative Bouin's solution contains picric acid, formalin, and acetic acid, which could also damage nucleic acid integrity [11]. Witchell et al. [6] suggested that, since formalin causes structural and chemical disturbances to nucleic acids, the use of another fixative (e.g., Bouin's solution) could improve the ability to use FFPE material for molecular studies. However, our present results showed that the use of Bouin's solution as a fixative was also associated with low-quality amplification.

While many variables involved in the RNA extraction process may be related to the amplification success, the exact role of each factor remains unknown. To investigate this subject, here we assessed the interference caused by quantity of RNA extracted, RNA purity assessed by the A260/280 ratio, the fragment size of the sample, the tissue type, the paraffin block storage time, the fixative types, and primers used for complementary DNA synthesis (random primer or oligo dT). Amplification quality was assessed by standard PCR to amplify the endogenous genes *β*-actin and* GAPDH* and by real-time PCR for* GAPDH*,* ABL1*, and* PRKG1*.

Hamatani et al. [12] and Scorsato and Telles [13] showed that the pH level interferes with PCR success and suggested that pH values between 6.5 and 9.0 are ideal for improving amplification efficiency. It is likely that the introduction of an additional washing step with PBS prepared with DNase/RNase-free water increased the pH of the solution, thus improving the quality of amplification by RT-PCR and conventional PCR. As fixatives are soluble in water, it is also possible that the residues of these substances were eliminated from the tissues through this washing step. As the addition of a washing step in protocol 2 improved amplification success with all types of fixatives, it appears that this step could remove the residual amounts of formalin, formaldehyde, and Bouin's solution. Similar results were achieved by Hamatani et al. [12], who found better PCR results after using lithium carbonate (Li_2_CO_3_) to discolor the paraffin-embedded material fixed with Bouin's solution prior to RNA extraction.

Our present results showed that the additional washing step in protocol 2 improved the extracted RNA concentration, as no RNA sample extracted via protocol 2 showed a concentration of less than 50 ng/*μ*L. We also found that RNA extracted with protocol 2 was of greater purity, as the whole samples produced a RNA purity ratio higher than 1.9. RNA extracted by protocol 2 also showed increased amplification rate from small fragments of ≤1.0 cm. Among these samples, amplification improved from 16.7% to 83.3 %. Thus, our present findings also suggest that the impact of the fixative on RNA extraction quality is greater for smaller fragments. In contrast to our data, Scorsato and Telles [13] did not find that the concentration and purity of RNA extracted from FFPE samples were correlated with amplification success.

We did not find that amplification was impacted by tissue type, block age, the primers used for cDNA synthesis, or the endogenous genes analyzed. However, our samples of different tissue types were heterogeneous, and thus further studies should be performed to confirm our present results. Our results concerning the age of the paraffin blocks were in accordance with those previously reported by Scorsato and Telles [13], who did not observe material loss or changes in RNA purity over time. They also demonstrated that agarose gel electrophoresis was not a sensitive tool for testing RNA quality. Therefore, here we analyzed RNA quality by both PCR and real-time PCR to check for differences in amplification success following both protocols. For both RNA extraction protocols, we observed higher amplification using real-time PCR compared to that with standard PCR. Using protocol 1, standard PCR resulted in amplification of 78.7% of samples, while real-time PCR resulted in amplification of 95.7% of samples. Using protocol 2, these amplifications rates were 87% with standard PCR and 93.5% in real-time PCR. The factors that interfered with amplification were the same in both methods.

Overall, our present results demonstrated that inclusion of a PBS washing step during the sample preparation for RNA extraction from FFPE samples produced a significant improvement in the RNA quality and in the success of the amplification by PCR and real-time PCR.

## Figures and Tables

**Table 1:** Factors related to PCR amplification from paraffin-embedded samples.

	Protocol 1		Protocol 2		*P* value
	Positive (%)	Negative (%)	Positive (%)	Negative (%)	
RNA quantity (ng/*μ*L)					
1–50	25.0	75.0	—	—	0.00025
50–100	37.5	62.5	33.3	66.7	
>100	70.4	29.6	89.3	10.7	
RNA purity level					
<1.7	0.0	0.0	0.0	0.0	0.0000001
1.7–1.9	16.7	83.3	0.0	0.0	
>1.9	62.5	37.5	83.9	16.1	
Sample size (cm)					
<1.0	16.7	83.3	80.0	20.0	0.034
1.0-2.0	71.4	28.6	80.0	20.0	
>2.0	75.0	25.0	100.0	0.0	
Fixative type					
Formalin	87.5%	12.5	100.0	0.0	0.000018
Formaldehyde	17.6%	82.4	73.7	26.3	
Bouin's solution	33.3%	66.7	80.0	20.0	

Protocol 1: RNA extraction using the Recover All Total Nucleic Acid Isolation Optimized for FFPE Samples kit (Ambion Inc., Austin, Texas, USA) following the manufacturer's protocol; Protocol 2: RNA extraction with the same kit with an additional PBS washing step; Positive: sample PCR amplification; Negative: sample showing no PCR amplification. With the chi-square and Mann-Whitney tests, a *P* value of <0.05 was considered significant.
